# Towards a midwifery profession in Bangladesh – a systems approach for a complex world

**DOI:** 10.1186/s12884-015-0740-8

**Published:** 2015-12-03

**Authors:** Malin Upper Bogren, Helena Wigert, Lars Edgren, Marie Berg

**Affiliations:** Institute of Health and Care Sciences, Sahlgrenska Academy, University of Gothenburg, Arvid Wallgrens Backe, Box 457, S-405 30 Gothenburg, Sweden; Centre for Person-Centred Care (GPCC), University of Gothenburg, Gothenburg, Sweden

**Keywords:** Midwifery profession, Midwifery, Complex adaptive systems, CAS thinking, CAS metaphor, Adaptive organisations, Coordination, South Asia

## Abstract

**Background:**

The midwifery profession is crucial for a functioning health system aiming at improved maternal and child health outcomes. Complex Adaptive Systems (CAS) can be used as a tool to understand actors’ interactions in the system around midwifery profession for improved maternal and child health. The purpose of this study is to explore how actors connect to promote the Bangladesh’s midwifery profession.

**Methods:**

An explorative study based on the framework of CAS was performed. Data were collected through semi-structured interviews with 16 key persons representing nine different organisations promoting the establishment of the midwifery profession. Qualitative analysis was used.

**Results:**

Findings show that the actors were intertwined and driving towards a common goal; to save lives through education and deployment of 3000 midwives. The unique knowledge contributions of everyone involved were giving the system strength and power to perform. Collaboration was seen as more could be achieved compared to what an individual organisation could do. Significant results of this were that two midwifery curricula and faculty development had been produced. Although collaboration was mostly seen as something positive to move the system forward, the approach to reach the set goal varied with different interests, priorities and concerns, both on individual organisational level as well as at system level. Frequent struggles of individual philosophies versus organisational mandates were seen as competing interests for advancing the national priorities. It would appear that newcomers with innovative ideas were denied access on the same terms as other actors.

**Conclusions:**

This study illustrates that CAS thinking can be used as a metaphor to understand how to adapt more emergent ways of working instead of the traditional planned approaches to change and develop in order to deal better with a more complex world. Through examining how actors connect for establishing a midwifery profession, offers insights of shared interests towards stepping up efforts for a competent midwifery profession in Bangladesh and elsewhere. Good relationships, where everyone’s expertise and innovations, are used to the full, are crucial for establishing a strong midwifery profession and thus improved maternal and child health.

## Background

Maternal health is a human rights concern to which child health is inextricably linked [1–3]. Though considerable global efforts have been made, maternal health targets show to be the hardest to achieve across the developing world and will take many years past 2015 to reach. A post-2015 agenda building on the millennium development goals is in progress, which stresses the importance of working in partnership to improve maternal and child health [4].

There is a growing agreement that midwifery care is the most effective solution to improve maternal health and subsequently child health [5, 6]. The ability of a midwife to demonstrate competence according to international standards [7, 8] and contribute to improving outcomes for women and newborns depends on various factors. These include the quality of education, license to practice, the regulated scope of practice, appropriately deployed and the existence of effective teamwork, referral mechanisms and with sufficient resources [5].

The returns of investing in professional midwives educated and regulated as per international standards are enormous, particularly in poor resource countries. According to 2014 series on midwifery in *The Lancet* [5, 9–11] midwifery care provided by midwives who are well educated, licensed and regulated, could prevent over 80 % of all maternal and neonatal deaths and stillbirths [10].

In an attempt to achieve such figures, Bangladesh with a population of around 165 million [12], is a country that have initiated efforts to educate midwives as per international standards, regulated by a regulatory body [13, 14]. Despite a 40 % decline in maternal mortality ratio in a period of nine years, 194 women are dying for every 100,000 live births, and one in 19 children die before reaching their fifth birthday. About 70 % of births take place outside health facilities and 68 % of all births are conducted without support by any skilled attendant such as midwives, nurses or doctors [12, 15].

All over the world, health care systems are becoming more complex; this also includes the health system for women and children. An effective collaborative approach among stakeholders in a health system is essential to achieve sustainable and equitable development of high quality health for women and children, especially in a low-income country [1]. A powerful tool to explore connections within a complex health system as well as to understand tension and conflicts concerning governance is through the theoretical framework of “Complex adaptive systems” (CAS) [16].

The purpose of this study is to explore how different stakeholders (or “actors”, to use CAS’s terminology) connect to promote the Bangladesh’s midwifery profession. CAS will be used as a tool to understand actors’ interactions in the system around the midwifery profession with the aim of improved maternal and child health.

## Methods

### Study design

Based on the theoretical framework of CAS [17–19], we have explored how actors around the midwifery profession in Bangladesh connect and relate to one another within the health system. We used CAS as a framework to describe and analyze the data, and to assess the theoretical fit of a CAS perspective with the dimensions that emerged in the interviewees’ responses. A CAS consists of several subsystems called actors, who self-organize and produce adaptations that emerge on ways that can neither be predicted nor controlled [20, 21]. A central aspect of a CAS is that actors need to work together to solve complex tasks. Research, education and health care systems are examples of such complex work [22, 23]. The necessary competence to perform a task is not owned by any actor, but is rather an outcome of interactions among the system actors. To achieve positive outcomes, each actor contributes with its own unique skills. How involved actors come together to tackle a common task is important to understand how development is progressing in different contexts [23].

Against this, our application of this framework focused on the connection between the actors promoting the establishment of a midwifery profession in Bangladesh. This framework was used to gain insight on how the system actors promoting the midwifery profession in Bangladesh are connected and come together in their establishment of the profession.

### Data collection

Data were collected in Bangladesh during April-May, 2013. Data sources were nine different actors involved in promoting the midwifery profession in Bangladesh. The term actor is here used for different departments within the government, university, professional association, Non-Government Organisations (NGO’s) and donors. The actors were clustered in five groups: government, university, professional association, NGOs and donors (Table 1).

**Table 1:** Respondents and actors

Actors	Respondents interviewed (*n* = 16)	Actors (*n* = 9)
Government	2	2
Academia	2	1
Professional Association	2	1
Donors	6	3
NGOs^a^	4	2

^a^Non-Government Organisations

First, relevant policy and education documents from the nine actors were collected and read through. Secondly, a semi-structured interview guide was developed, with open-ended questions in relation to four key areas: organisation and its resources, collaboration, communication channels and future plans [19].

Third, key persons representing the actors were purposively selected [24] in relation to their positions and policy influence in their respective organisation/actor. Digital voice recorded individual interviews were performed with sixteen key persons representing these actors (Tables 1 and 2) All persons were informed about the aim of the study procedures, and confidentiality was assured.

**Table 2:** Characteristics of the respondents (*n* = 16)

Gender	Female	14
	Male	2
Age range		41–67
Median		48
Profession	Nurse	1
	Nurse-Midwife	9
	Medical Doctor	5
	Other	1
Academic qualification	Diploma	1
	BSc	2
	Master’s	10
	PhD	3
No. of years employed within the current organisation	2–7	6
	8–15	5
	16–23	5

All interviews were conducted in English, and lasted about 40–60 min each. Many times the interviews were interrupted due to someone entering the room. The interviews could despite this continue without any significant disruption, as respondents were familiar with this situation. The respondents were encouraged to speak freely and probing questions were asked on aspects related to the study purpose. Fourteen of the interviews took place at the workplace of the respondent and two at a social center chosen by the respondents.

### Data analysis

The interviews were transcribed verbatim. Qualitative data analysis was performed inspired by the work of Miles, Huberman and Saldana [25]. First the transcripts were read several times in order to get a sense of holistic impression. Next, the text data were analysed through three concurrent flows of activities performed as a continuous, iterative enterprise: data condensation, data display, and conclusion drawing and verification [25].

#### Data condensation

The first flow aims at selecting, focusing, and simplifying the transcript data. Initially the text of each interview was condensed in order to contain only information relevant to the purpose of the study. Second the text data were coded, dividing the content into parts, with the purpose of making it possible to identify content characteristics on a more abstract level. Codes reduced large amounts of data into smaller numbers of analytic units.

#### Data display

In the second flow of analysis, data were organised and compressed to make the description of the study phenomenon sharp. The codes were imported into a designed matrix where the rows and columns represented each of the sixteen interviews. In the analysis pattern, meanings were clustered, and successively essential structures emerged that describe and explicate how actors within midwifery education, regulation and association collaborate to promote Bangladesh’s midwifery system at its operational level.

#### Conclusion drawing and verification

The third flow of analysis involves testing the meaning that emerges from the data for their likelihood and for whether or not they can be confirmed. To ensure validation and reach a final conclusion, all of the authors (MUB, MB, LE, and HW) made separate analyses, which were discussed until common agreement on codes and final themes were achieved.

### Ethical considerations

The responsible research body for the study was University of Gothenburg. According to Swedish rules and guidelines for research [26] and Bangladeshi rules and guidelines for research, at the time of data collection, no ethical approval was necessary since no patients were involved, nor were health care staff in relation to service provision. Permission to perform this study was obtained by the manager responsible for each organisation (governments, universities, professional association, NGO’s and donors) which has been part of the study [27]. The study was carried out in accordance with Swedish Law and the Declaration of Helsinki [28]. To protect and respect the confidentiality of these organisations and the individual respondents no details are being mentioned. Approval was obtained through a signed consent form by all respondents prior to the interview. All respondents were informed verbally and received written information reflecting the research objectives. They were invited to ask questions about their participation and were made aware that participation was fully voluntary, anonymous and that they could withdraw without explanation at any time. The benefits of interviewing selected key persons in recognized organisations are that these have extensive experience from inter-organisational collaboration. The interview data represented personal opinions and do not necessarily stand for the values of their respective organisation. This is, however, not a risk as no objective truth was searched for.

## Results

The analysis of data generated five general dimensions, describing how actors connect to promote Bangladesh’s midwifery profession. Each dimension is presented below. Quotations from the 16 respondents are labeled R1-R16.

### Having a common goal

A common goal for all actors was to save lives, i.e., reduce maternal and child mortality and morbidity ratio. This was the driving force to promote the Bangladesh midwifery profession to respond to the national challenge. A breakthrough for stepping up efforts towards reaching these goals was the “Strategic Directions” from the government in 2008 for enhancing and utilization of nurse-midwives [14]. These directions were developed with support from donor 1 and 2.

The need to educate qualified professional midwives as per international standards was identified as crucial in saving the lives of women and newborns. All actors stressed that this was a first benchmark for the midwifery profession framework and also one of the first collective system achievements towards a separate midwifery profession.

*The Strategic Directions lift the current development and really supported the government in moving forward with the midwifery profession in Bangladesh.* (R13)

*With the support of donor organisations we have developed Strategic Directions for the midwifery services to increase the production of midwives.* (R9)

The progress for stepping up these efforts gained momentum after a speech held by the Prime Minister at the General Assembly of the UN in 2010, where she committed to the education and deployment of 3000 midwives fulfilling international standards by 2015. This clear statement added fuel to the system and contributed to joint efforts to achieve the set target. In general, a strong task determination was confirmed among all actors, knowing that an uncertain world requires flexible and appropriate responses to identified needs. The ability to transform the 2008 Directions into opportunities for higher education and to produce positions for midwives is depending on detailed agreements for how it will be achieved.

The actors stated that there is no consensus being made for how to advance the progress. The importance of having clear objectives and interventions, translated into firm commitments for collaborative actions was stressed, to ensure that only one line for growth of the profession is to be followed: the one decided by the government. The government actors and the professional association stated that they do not have the capacity to take such an initiative, though the multi -and bilateral actors keep a low profile and instead try to support the government to take the leading role for owner and stewardship in policy-making.

*If you want to push for an issue, then everybody needs come together in order to push for the same issue. If different people are pushing for different midwifery issues, then the chances are that none of those issues will happen, but if you make a priority list on what exactly needs to be done and by whom, it is more likely to push the agenda forward.* (R4)

### Contribute with different competencies

To achieve the common goal within the system, the actors connected with their different competences, which nurtured the system promoting Bangladesh’s midwifery profession. To nurture the system means that each actor contributes with its own unique competence. A broad range of competencies were identified (see Table 3). The actors’ unique competence included specific knowledge, skills, behavior and attitudes. All together the competencies included necessary competencies in a chain of change, from advocacy to policy change to make final policy decisions. None of the competencies alone will establish a midwifery profession, but as a joint contribution they were giving the system strength and power to perform.

**Table 3:** Each actor’s unique competence contributing to promote Bangladesh’s midwifery profession

Government 1	Decision makers for regulation and service delivery
Government 2	Decision makers for regulation and education
University	Implementers of education
Professional Association	The voice of midwives
NGO 1^a^	Advocacy for policy change
NGO 2^a^	Innovation
Donor 1	Technical and financial support including coordination on multi-lateral level
Donor 2	Technical and financial support including coordination on multi-lateral level
Donor 3	Technical and financial support including coordination on bi-lateral level

^a^Non-Government Organisations

*Technical assistance contributes to a broader knowledge about who the midwife is, what she does, what her competencies are and also the regulation around midwives who have undertaken education. Technical assistance also contributes to how to develop a midwifery association to support the profession of midwives.* (R5)

An ingredient that connected the system actors was the notion of knowledge sharing. The perception was that by taking advantage of each other’s competencies and expertise, all actors would gain mutual understanding on how to tackle common challenges. Utilising the expertise within the system to the full was seen as an efficient way to collaborate.

*Everything is for the improvement of the Bangladesh situation, so we have to work together and share each other’s knowledge and making the best out of it, then I think that we will be able to succeed*… *the knowledge and experience of everyone should be brought together.* (R11)

All actors noted the importance of competence, and the contributions of all actors were required in order to have an essential set of competencies to achieve the goals. This created a dynamic thriving system in which the actors were interdependent on each other’s resources to deliver.

### Move forward through collaboration

To move forward towards reaching the goal; to save lives through the establishment of qualified midwives, collaboration was highlighted as essential by all actors. A prerequisite for collaboration was tight relationships and interactions. The driving force was the alignment of shared interests around the commitment to train and deploy the 3000 midwives. Through collaboration, more could be achieved compared to what an individual organisation could do:

*All women have the rights to have a safe pregnancy and safe delivery, and children have the rights to survive, this is the priority. To progress further, the country needs midwives. We need all actors to collaborate. If there is one agency, ministry or stakeholders who don’t understand the importance, nothing will happen.* (R14)

*It is impossible to work alone, it is a big job and it is not possible, what else can I say.* (10)

As a product of collaboration within the education pillar, several activities have taken place to initiate midwifery education on different levels. A six months midwifery curriculum for the existing nurses and a three year direct-entry curriculum on a diploma level have been developed. Similarly a written development plan for faculty (teachers) has been produced, and training of teachers has started (by seven actors; NGO 1 and 2 did not participate). In addition, international exposures, effective advocacy and media campaigns have taken place (by eight actors; NGO 2 did not participate). It was expressed that:

*None of these accomplishments would have happened unless of collaboration within the system, as well with support from international expertise jointly funded by the two multilateral actors.* (R 8)

A critical role of how the different actors connected to one another was the level of relationship-friendship established. The building blocks for successful activities are depending on personal relationships within the system. Personal relationships were perceived as enabling but also a hindering ingredient. Among the actors where collaboration worked out less positively, disagreements were on an individual level rather than on the system level. This was expressed as “*feeling blackmailed and being threatened*”. (R15)

#### Challenges to collaborate

Collaboration was mostly seen as something positive to move the system forward. While this may sound simple, the system’s reality was more complex. The approach to reach the set goal varied with different interests, priorities and concerns, both on individual organisational level as well as at system level. Frequent struggles of individual philosophies versus organisational mandates were seen as competing interests for advancing the national priorities.

*All organisations still want to do their own things. There is a pressure from headquarters which we report to.* (R14)

*Each and every organisation has its own roadmap of actions.* (R6)

For instance, the two government actors had to follow the culture of the government; constantly working hard in an environment of lack of resources and political unrest, while the donors had their own mandate and processes to follow, which were perceived by some other actors as a branding issue and as a result it turned out to be a personal agenda. For example:

*Organisations make it a branding issue because they think it is a personal agenda. If we want to move forward we have to come together and need to remove our organisational hats, and work for the national agenda. We need to get together, prioritise and stay focused and proceed step by step.* (R4)

Contributing factors to the challenge to collaborate were linked to different issues. For example it was expressed that the government actors did not take enough ownership. On the other side governmental actors did see themselves as underprivileged.

*We need to be stronger, we need more support, we* don’t *have a car so I can’t move with my team, we need more manpower, computers and more space, and this is an urgent need for us.* (R10)

Another challenge was that the NGOs were reflected by other actors as important, but considered as newcomers to the system and thus could not yet fulfill the required professional expertise. For example, their lack of capacity was expressed in relation to training midwives according to international standards, as illustrated in the following quote:

*I don’t know exactly how they work; they are not very transparent in their way of working. They have developed their own midwifery curriculum which did not meet international standards. Through a lot of advocacy work; this has now been changed, but it is still not the same as the national curriculum. It would be better if it was harmonised.* (R5)

Thus, as not being fully accepted, the NGO 2 had to develop other strategies. To get around these challenges, NGO 2, with its larger freedom chose to go their own way to move more rapidly forward by introducing their own programme. With external resources and international academic collaborative partners, they have created their own separate educational system for midwives. They have also created positions for the new midwives. After completion of education, all newly educated midwives were deployed with the title midwife within the NGO 2’s health facilities.

**Fig. 1:**
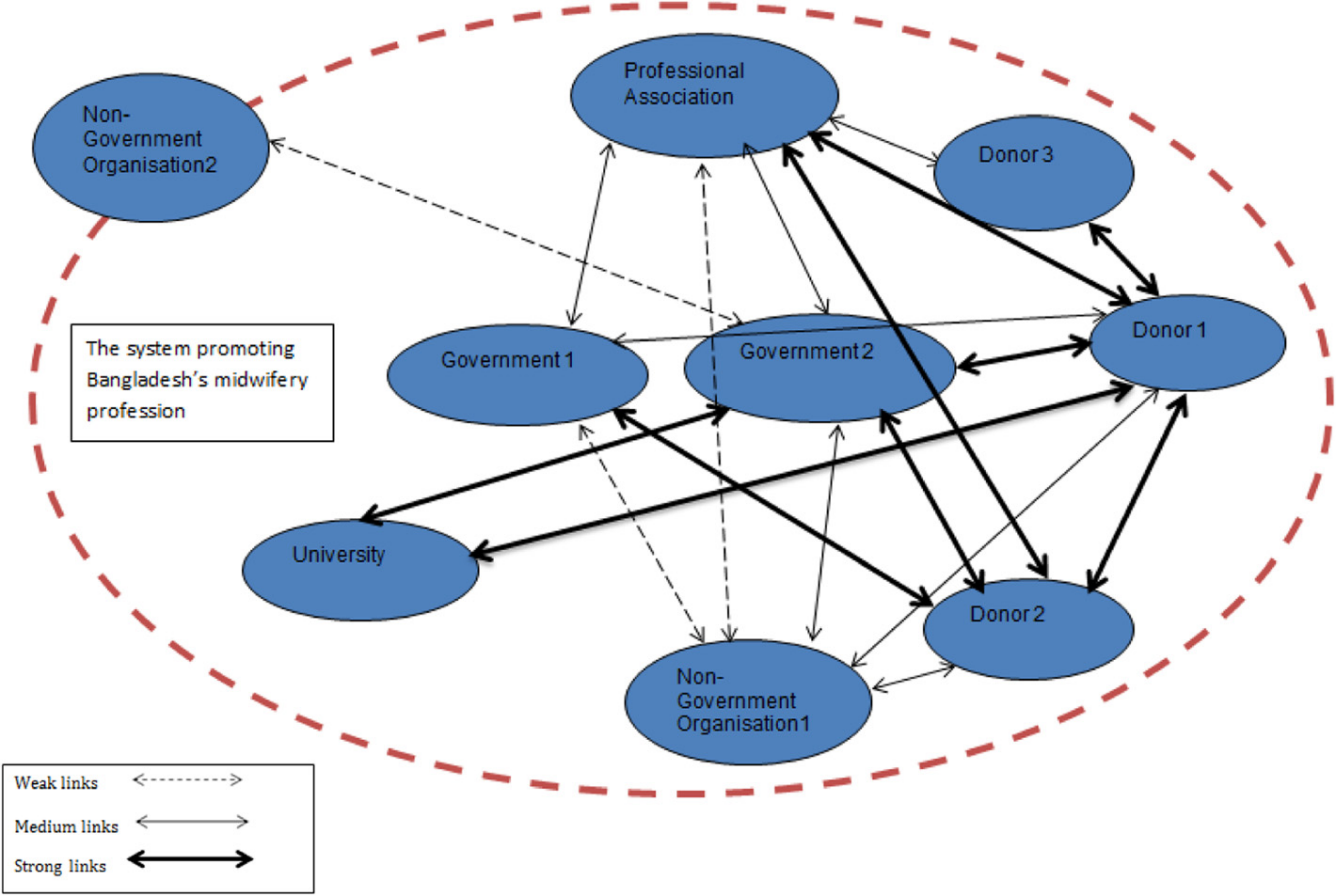
Illustration of the collaborative links between actors promoting Bangladesh’s midwifery profession

To summarize the collaboration between the nine actors; it appears that some collaborated more, and strong links were developed, while others had weaker links. The reasons for stronger links may be related to interdependency or dependency. While others, such as the NGOs, were not fully accepted or were accepted on different levels. See Fig. 1.

Another example of competing interests that was a hindering factor for effective collaboration is that representatives from Government 1 and Government 2 expressed the need to align their respective responsibilities as they worked with similar activities. Nevertheless, in reality, financial and technical support created competing interests between the two actors as one of them received more financial support than the other. This unequal financial support to the two different governmental authorities was counterproductive to effective collaboration, as expressed in the following quotation:

*Education and regulatory activities are not being worked on simultaneously and fewer resources are provided for deployment as the donors are working more closely with other authorities, but I think they should work with us… if we don’t receive logistics and funds, it is difficult for us to fulfill our mandate.* (R 9)

### Create communication channels for visibility

The mode of communication was a central part within the system of actors promoting the midwifery profession. Both formal and informal communication was mentioned as essential. A list of the different communication channels mentioned by the respondents is illustrated in Table 2. The communication channels varied through a mixed way of communication depending on whether the communication was personal or official.

The communication tools used for personal one-to-one communication were emails, text messages, phone calls and social networks. For official mode of communication, letters were used to invite to face to face meetings with donors, policy-makers and key stakeholders. To reach out with a broader general communication a more interactive approach was applied, such as field visits, group discussions, social networks, advocacy events e.g., celebration of International Day of Midwives.

#### Challenges to communication

There were identified challenges to communication within the system. The government actors found it challenging to maintain time to communicate due to scarcity of manpower and shortage of electricity. With poor internet access and lack of computers, they felt they were behind in technology as illustrated in the quote below:

*It is very difficult to communicate, mostly we need to use our personal mobile phones, and we don’t have internet access all over the country. Other places have internet but not full time electricity. Due to lack of electricity it makes it problematic to communicate. (R9)*

Email was considered as a common mode for communication, though the opportunity to read emails was poor and was not used as much as official letters and phone calls. The NGO actors found that communication was not always transparent enough and saw the communication more as a need and demand based activity, and preferred face-to-face meetings and round table discussions for true dialogue and clear communication. All actors agreed that communication was a critical factor to maintain the collaboration. Opportunities to communicate on equal levels varied among actors, (Table 4), and information did not always reach the government actors for timely actions.

**Table 4:** Mode of communication channels among the actors promoting Bangladesh’s midwifery profession

Mode of communication	Government	Professional associations	Non- Governmental	University	Donors
Official letters	√	√		√	√
E-mail	√	√	√	√	√
Social media		√			
Face-face meetings	√	√	√	√	√
Phone calls	√	√		√	√
Conferences					√
Seminars/Round table		√	√		
Task force meeting				√	√
Retreats					√
Field visits	√		√	√	√
Advocacy events	√	√	√	√	√
Electronic conferences			√		√

### Being dependent on financial and technical support

**Fig. 2:**
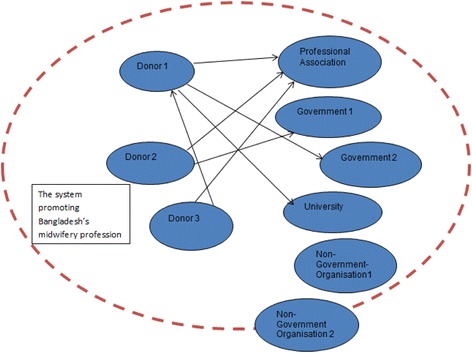
Illustration of financial support links within the system promoting Bangladesh’s midwifery profession

**Fig. 3:**
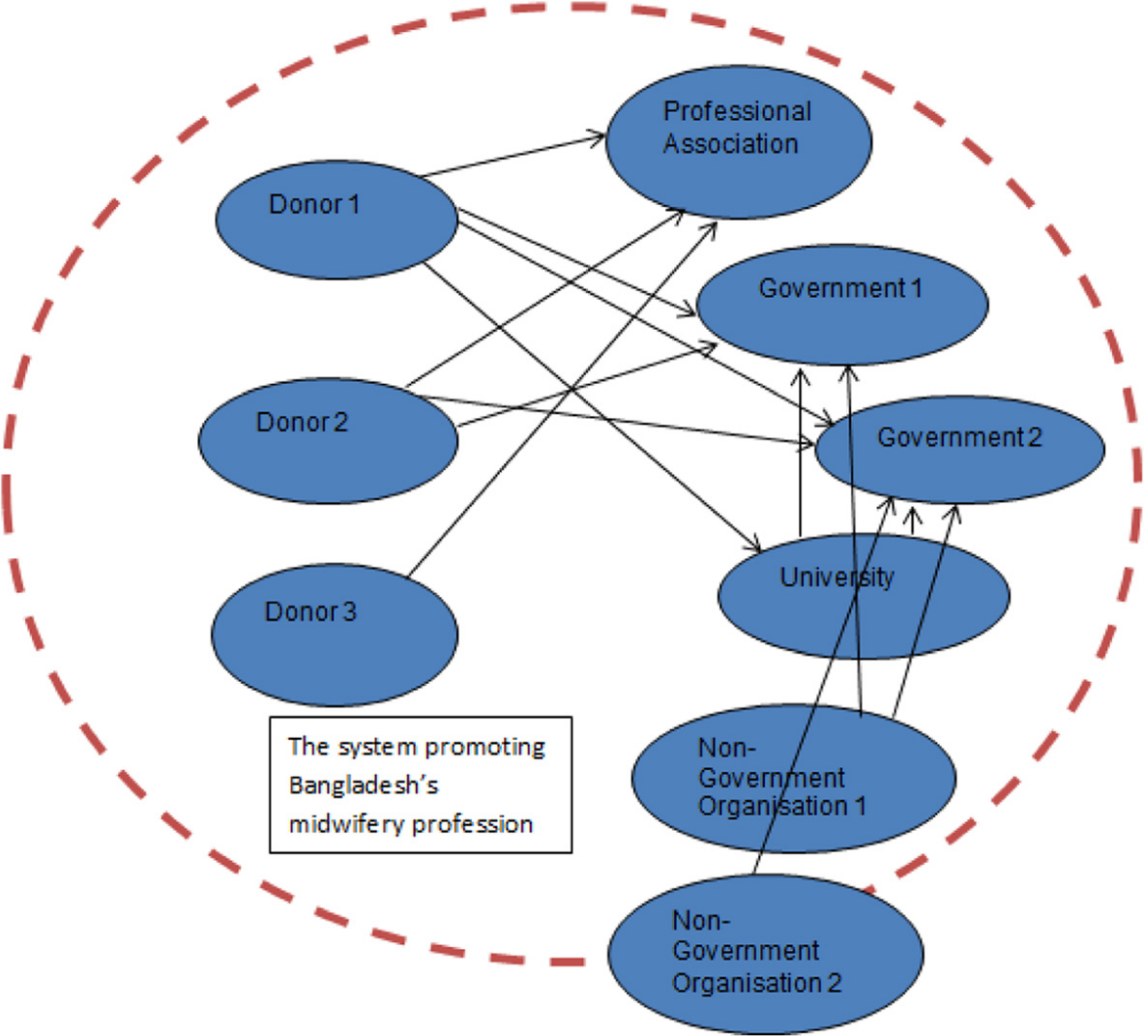
Illustration of technical support links within the system promoting Bangladesh’s midwifery profession

Three actors gave financial support, and one of these got indirect financial support from another. All donors gave financial support to the professional association. All donors provided technical support to all actors except to the NGOs. NGO 1 supported the government actors with technical support. It became clear that the donors provided most of their technical and financial support to the education pillar. Details are illustrated in Figs. 2 and 3.

The birth of the professional association actor was a result of external financial and technical support. This actor is still young and is struggling to stay viable, trying to be an unquestioned part of the system and to keep its place.

*We can’t do anything independently to reach the goal. We as an organisation need education, we need technical support, we need more skills, we are learner and for that reason we need support from other organisations. If we need to work faster, we need to work together. (R2)*

The government actors play a vital role in promoting the midwifery profession, but considered themselves as fragile bodies, as they were in need of extensive financial and technical support to become stronger in order to achieve the commitment to train and deploy 3000 midwives. Crucial for retaining the educated midwives was that positions for midwives were created and at the time there were no positions. Therefore the newly educated midwives went back to their nursing positions and provided the same services as before graduation as midwives. As a result, the educated midwives’ contribution to the declining maternal and infant mortality rate could not be evaluated (as they were categorised as nurses). Consequently, as expressed by R9: “*we did not receive any positive feedback for our hard work”.*

## Discussion

The findings illustrate how actors from 2008 onwards were intertwined and driving towards a common goal; to save lives through education and deployment of 3000 midwives. The unique knowledge contributions of everyone involved were perceived as self-evident without being questioned, and were giving the system strength and power to perform. Curriculum and faculty development, international attention and media campaigns are significant results of collaborative approaches. It would appear that newcomers with innovative ideas were denied access on the same terms as other actors. Therefore the actions of an innovative newcomer came as a surprise and became a warning bell that shook the system and seemed to be hampering the system seriously. This is in accordance with CAS theory, which says that actions are non-linear, meaning that even small changes can have large effects. This is called the butterfly effect [29]. Some important lessons are discussed in the following sections, particularly in relation to CAS theory.

Findings show that a driving force to promote the Bangladesh midwifery profession was the unified goal to save lives through educating more midwives. In order to make this true, the actors expressed a need for having a joint implementation plan with a clear vision, based on the 2008 Strategic Directions. The system would consequently grow more powerful with an ability to influence the broader health system. Balabanova et al. [30] and Campbell et al. [31] argue that partnership between government and non-governmental actors are factors connected with successful development of important health policies. We suggest that, translating the 2008 Direction into clear and simple rules including an evaluation giving important feedback of the working process, would engage all actors and further advance the execution of the 2015-goal of 3000 trained midwives.

A prerequisite for the actors promoting Bangladesh’s midwifery profession completing its mission to train 3000 midwives in a short time was the coming together and contributing different unique competencies. Despite the heterogeneous composition of actors, the way the existing system works does not allow fruitful relationships with newcomers to contribute to their important work. Newcomers are needed for successful strengthening of the midwifery profession in Bangladesh, as they have a unique system competence that manifests itself and can relatively quickly launch a functioning system. The system is kept together and evolves as interdependent, as no actors are able to handle the mission of the system on its own [20]. The purpose of the system is to create optimal conditions for the actors when working together in their tasks [21]. To position and profile its mandate, the actors promoting Bangladesh’s midwifery profession need to engage all actors in discussions around their existence, through developing clear and simple rules to act as guidance. Dodder and Dare [32] talk about simple, fundamental principles. Their study has shown that these principles are essential for re-installing vitality, and not for actors to drown in procedural matters. This requires an open and inviting attitude between all involved actors.

Findings show that collaboration, tight relationships and close interactions were all factors essential to move closer towards the common goal. As a result of this, secured funding for capacity building in terms of pre-service and in-service education was made. According to Chinnis and White [33] connection and relationships between actors are two critical aspects for a system to survive. The relationships between actors are seen as more important than the actors themselves. In the studied midwifery system, the connectivity among the actors appears loosely coupled and their “organisational hats” are kept on. Organisational mandates and individual viewpoints are consequently followed, instead of the system interests. In order to maintain the system, Edgren and Barnard [21] state that individual organisational interests need to be put aside in favor of the system, but to do so, trust and respect need to be nurtured.

NGOs are recognised as having the ability to achieve national health outcomes. There are, however, differences in basic institutional approaches and structures between the government and NGOs. Ideological differences have been identified and consequently a negative governmental attitude developed towards NGOs. In order to avoid the government authority culture and having their organisational freedom affected, NGOs many times chose to go their own way [34]. This is, as mentioned earlier, true for one of the actors in this study: NGO 2. With large operational expertise in the health sector, NGO 2 had the capability to act on its own; nevertheless this is not the ideal way to progress in the future. While its programme appeared to be successful, this is an example of system fragmentation, i.e., parts of a system act on their own without appreciating the whole [35–37]. We, therefore, suggest that the government needs to take the lead and invite NGOs to health programming discussions. For the survival of the system it is important that the actors stick together.

Actors promoting professional midwives in Bangladesh are facing pressure to intensify their relations to work more closely together to achieve high set goals. Nevertheless, this requires clear and ongoing communication. Dawson et al. [38] argue that clear communication is one of the characteristics to maintain collaboration. Our results show there is a limited communication capacity within the Bangladesh government’s inertia infrastructure, compared to the other actors. As shown in Table 4, there are, for instance, different prerequisites for mode of communication channels. As an example; the government lacks electronic devices, electricity and human resources, which make it a challenge to read and respond to electronic communication. As a result, this may be interpreted by others as lack of ownership and commitment.

The system is dependent on financial and technical support and would consequently not function without it. The donor actors are important providers of this support. However, instead of only providing support, a combination of support and strengthening would be a beneficial alternative. In agreement with Chee et al. [39], with support from the donor actors, the design of effective strengthening of interventions would improve multiple health services with long-term impact such as helping the system function well and not just filling gaps. Strengthening initiatives such as strengthening legal recognition of the profession through provision of a regulatory framework and a deployment system [6, 40, 41], would be initiatives that could add value to the already developed education pillar. Consequently, these initiatives would contribute to the government fulfilling its commitment to provide a high quality, equitable standard of maternal health care.

### Strengths and limitations of the study

The key strength of this study is that it is contributing to how the principles of CAS theory can be applied in empirical research. Our findings suggest that CAS perspective is a useful framework for guiding the development and establishment of the midwifery profession in low-income countries. Using interviews as a data collection method allowed the respondents to express their perspectives freely about how they connect to promote the Bangladesh’s midwifery profession. The findings of this study are, however, restricted to individuals representing different organisations, and may not necessary stand for the values of what their organisations represent.

## Conclusion

There are three learning experiences with which to conclude. First, the way a system accepts an incoming innovator from the outside who wants to contribute. Second, the process of internalising a new mission, and third, whether or not there will be anyone giving altruistic support in hard times. The future will show whether this CAS can handle the challenges ahead. This study illustrates that CAS thinking can be used as a metaphor to understand how to adapt more emergent ways of working instead of the traditional planned approaches to change and develop in order to deal better with a more complex world. The knowledge gained from this study could be used in other countries that are beginning to design programmes for a midwifery cadre based on a supported regulatory framework.
